# Room-Temperature Thermoelectric Performance of n-Type Multiphase Pseudobinary Bi_2_Te_3_–Bi_2_S_3_ Compounds: Synergic Effects of Phonon Scattering and Energy Filtering

**DOI:** 10.1021/acsami.3c01956

**Published:** 2023-04-04

**Authors:** Sima Aminorroaya Yamini, Rafael Santos, Raphael Fortulan, Azdiar A. Gazder, Abhishek Malhotra, Daryoosh Vashaee, Illia Serhiienko, Takao Mori

**Affiliations:** † Department of Engineering and Mathematics, Sheffield Hallam University, Sheffield S1 1 WB, U.K.; ‡ Materials and Engineering Research Institute, Sheffield Hallam University, Sheffield S1 1WB, U.K.; § Australian Institute for Innovative Materials (AIIM), University of Wollongong, North Wollongong, New South Wales 2500, Australia; ∥ Department of Materials Science and Engineering, North Carolina State University, Raleigh, Raleigh, North Carolina 27606, United States; ⊥ International Centre for Materials Nanoarchitectonics (WPI-MANA), National Institute for Materials Science, Tsukuba 305-0044, Japan; # Graduate School of Pure and Applied Science, University of Tsukuba, Tsukuba 305-8577, Japan

**Keywords:** multiphase, thermoelectric, energy filtering, phonon scattering, bismuth telluride-based

## Abstract

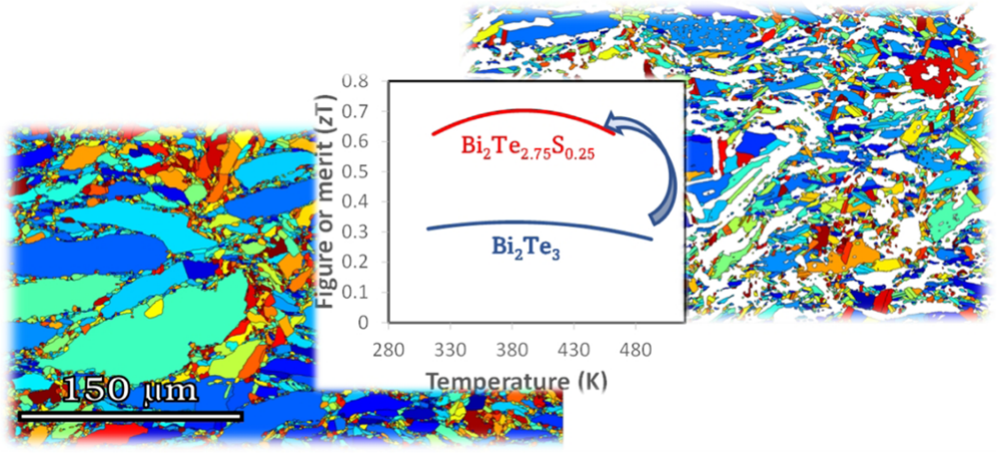

Bismuth telluride-based alloys possess the highest efficiencies for the low-temperature-range (<500 K) applications among thermoelectric materials. Despite significant advances in the efficiency of p-type Bi_2_Te_3_-based materials through engineering the electronic band structure by convergence of multiple bands, the n*-*type pair still suffers from poor efficiency due to a lower number of electron pockets near the conduction band edge than the valence band. To overcome the persistent low efficiency of n-type Bi_2_Te_3_-based materials, we have fabricated multiphase pseudobinary Bi_2_Te_3_–Bi_2_S_3_ compounds to take advantages of phonon scattering and energy filtering at interfaces, enhancing the efficiency of these materials. The energy barrier generated at the interface of the secondary phase of Bi_14_Te_13_S_8_ in the Bi_2_Te_3_ matrix resulted in a higher Seebeck coefficient and consequently a higher power factor in multiphase compounds than the single-phase alloys. This effect was combined with low thermal conductivity achieved through phonon scattering at the interfaces of finely structured multiphase compounds and resulted in a relatively high thermoelectric figure of merit of ∼0.7 over the 300–550 K temperature range for the multiphase sample of n-type Bi_2_Te_2.75_S_0.25_, double the efficiency of single-phase Bi_2_Te_3_. Our results inform an alternative alloy design to enhance the performance of thermoelectric materials.

## 1 Introduction

Two-thirds of the world’s energy is wasted as heat, with low-grade waste heat, up to 500 K, accounting for 60% of this lost energy.^1^ Thermoelectric generators (TEGs) that convert thermal energy to electricity and vice versa are a pioneer recovery technique for low-grade waste heat; several promising materials have been explored for power generation applications including GeTe,^2^ PbTe,^3^ and silicide.^4^ However, there are only a few thermoelectric materials that perform relatively well in TEGs within this temperature range, with a conversation efficiency of <10% over a narrow temperature range.^5^ Therefore, further development is required to improve the conversion efficiency of current thermoelectric materials, defined by the dimensionless figure of merit, , where α, σ, κ_e_, and κ_l_ are the Seebeck coefficient, the electrical conductivity, the electronic thermal conductivity, and the lattice thermal conductivity, respectively.

The state-of-the-art thermoelectric material for the low-temperature range (<500 K) is based on Bi_2_Te_3_ alloys. Despite significant advances in the conversion efficiency of p-type Bi_2_Te_3_-based materials, upon reaching *zT* of ∼1.5 at room temperature,^6,7^ the n*-*type pair still suffers from poor efficiency, and the *zT* of ∼0.9 is still the highest value at room temperature,^8^ resulting in low generated power by the TEG. This is associated with the lower number of electron pockets near the conduction band edge of Bi_2_Te_3_ than the valence band, failing to take advantages of the power factor, *S*^2^σ, enhancement through engineering the electronic band structure by convergence of multiple bands.^9^ Therefore, a new approach is required to tackle its persistent low *zT*.

Recently, multiphase thermoelectric materials have attracted the attention of the thermoelectric research community^10^ due to higher degrees of freedoms that these materials provide to design high-performance compounds through phonon scattering at interfaces,^11,12^ energy filtering,^12,13^ modulation doping,^14,15^ and potentially benefiting from incorporating magnetic interaction.^16,17^ The energy filtering effect creates potential barriers in the electronic band structure of the matrix through interfaces with the secondary phases, resulting in an increase in the overall Seebeck coefficient.^13,18^ The secondary phases with the larger band gaps and higher carrier concentrations than the matrix can increase the electrical conductivity of the multiphase compounds called modulation doping.^15,19^ These strategies are adopted to improve the thermoelectric performance, mostly by enhancing the power factor (α^2^σ); however, the lattice thermal conductivity is influenced at the presence of secondary phases.^17,20^

Here, we have selected a pseudobinary Bi_2_Te_3_–Bi_2_S_3_ system, which contains multiphase compounds of Bi_2_Te_2_S and Bi_2_Te_3_ or Bi_2_S_3_ phases. Both phases of Bi_2_Te_2_S (∼0.28 eV)^21^ and Bi_2_S_3_ (1.3 eV)^22^ have higher band gaps than Bi_2_Te_3_ (∼0.15 eV).^23^ We have shown that n-type multiphase pseudobinary Bi_2_Te_2_S–Bi_2_Te_3_ compounds possess a higher power factor and simultaneously provide lower total thermal conductivities than single-phase counterparts, resulting in a relatively high thermoelectric figure of merit of ∼0.7 over the temperature range of 300–550 K, compared to ∼0.3 for the Bi_2_Te_3_ sample. These new findings will inform the design of next-generation, multiphase compounds with enhanced thermoelectric performance for low-grade waste heat recovery applications.

## 2 Materials and Methods

### 2.1 Synthesis

A set of polycrystalline Bi_2_Te_3–*x*_S_*x*_ (*x* = 0, 0.25, 0.5, 0.75, and 1.0) samples were synthesized by direct reaction of stoichiometric amounts of high-purity Bi (99.999%, Alfa Aesar), Te (99.999%) shots, and dried S (99.99%) powder in vacuum-sealed quartz ampoules in an Ar glove box. The ampoules were homogenized at 1123 K for 10 h, quenched in cold water, and annealed at 673 K for 72 h. The obtained ingots were hand-ground to fine powders in an agate mortar and pestle housed inside an Ar atmosphere glove box. The powders were then loaded into a graphite die and sintered under vacuum to produce rods of 6 mm in diameter and 12–14 mm in length, using spark plasma sintering at 633 K and an axial pressure of 50 MPa for 5 min. The density (ρ) of samples was measured via the Archimedes method such that the relative densities were ≥99% for all samples.

The multiphase samples of  (*y* = 0.005, 0.01, and 0.02) that are iodine-doped samples of multiphase Bi_2_Te_2.75_S_0.25_ were fabricated to ingots by direct reaction of stoichiometric amounts of high-purity Bi (99.999%, Alfa Aesar), Te (99.999%) shots, dried S (99.99%) powder, and BiI_3_ (99.999%, Alfa Aesar Puratronic), using the abovementioned heat treatment procedure. The hand-ground powders were then loaded into a graphite die and sintered under vacuum to produce pellets using spark plasma sintering (FCT System GmbH, KCE FCT-H HP D-25 SD, Rauenstein, Germany) at 673 K and an axial pressure of 50 MPa for 5 min, obtaining samples with 11 mm in diameter and ∼12 mm height. Although the same sintering parameters were used to sinter these samples, the change in the SPS equipment resulted in samples with densities (ρ) of >90% of the relative densities, lower that the densities of >99% of the theoretical density for previous samples.

### 2.2 Material Characterization

The phase purity and crystal structure of sintered samples were characterized by powder X-ray diffraction (XRD) using a PANalytical X’Pert PRO X-ray diffractometer with Cu Kα radiation (λ = 1.5406 Å, 40 kV, 25 mA). The lattice parameters were determined by Rietveld refinement of the collected diffraction patterns using the FullProf program.

For the microstructure and chemical mapping, samples were cut from the sintered disk cross section of Bi_2_Te_3–*x*_S_*x*_ (*x* = 0 and 0.5) ingots such that the compression direction was parallel to the sample surface. The sample surface was polished up to 1 μm diamond and then subjected to Ar-ion milling on a Leica EM RES101 at 4 kV for 1 h. The grain and phase distribution and chemical composition of samples were characterized using a JEOL JSM-7001F field emission gun scanning electron microscope (SEM), equipped with an Oxford Instruments 80 mm^2^ X-Max energy-dispersive spectroscopy (EDS) detector and a Nordlys-S(II) electron backscattering diffraction (EBSD) detector working concurrently with the OI Aztec acquisition software. Combined EBSD and EDS maps were collected from 480 × 360 μm^2^ areas at an accelerating voltage of 15 kV, a probe current of ∼5.5 nA, and a step size of 0.24 μm. In the Bi_2_Te_3_ and Bi_2_Te_2.5_S_0.5_ samples, overall indexing rates of ∼91.5 and 92.2% were obtained such that most zero solutions were located at grain boundary regions. The maps were cleaned by removing wild orientation spikes and filling-in zero solutions via cyclic extrapolation down to five neighbors and processed using the OI HKL Channel-5 analytical software suite. The parameters used to collect and process the combined EBSD and EDS maps are detailed in our previous study.^24^

### 2.3 Electronic Transport Property Measurements

The electrical conductivity and Seebeck coefficient were measured perpendicular and parallel to the compression/sintering direction of the pellet and rod samples, respectively, by cutting ∼2 × 2 × 9 mm^3^ specimens from the pellet. The measurements were carried out from room temperature to 523 K under a helium atmosphere by using a Linseis LSR-3 apparatus.

The laser flash diffusivity method (Linseis LFA 1000) was used to measure the thermal diffusivity of the pellet and rod samples. The thermal diffusivity (*D*) of all samples was measured parallel to the compression/sintering direction. The thermal conductivities (*κ*) were calculated by κ = *D* × *C*_p_ × ρ. The heat capacity (*C*_p_) of all samples was measured according to the ASTM-E1269-11 standard test using a differential scanning calorimeter (PerkinElmer-DSC 8000). The density (ρ) of samples was measured via the Archimedes method.

The samples were cut with their long axis perpendicular to the sintering direction of samples to measure the Hall coefficient (*R*_H_) from transverse electrical resistivity measurements using the DC transport option of a Quantum Design physical property measurement system (PPMS) at temperatures between 5 and 400 K under a ±2 T magnetic field. The Hall carrier concentration (*n*_H_) and Hall carrier mobility (μ_H_) were calculated using *n*_*H*_ = 1/(*R*_H_·*e*) and μ_H_ = σ/(*n*_H_·*e*), respectively. The room-temperature Hall coefficient (*R*_H_) of doped samples was measured through van der Pauw method, using an ECOPIA-HMS-3000 apparatus to obtain the room-temperature Hall carrier concentrations and electronic mobilities.

## 3 Results and Discussion

### 3.1 Composition and Microstructure Analysis

**Figure 1:**
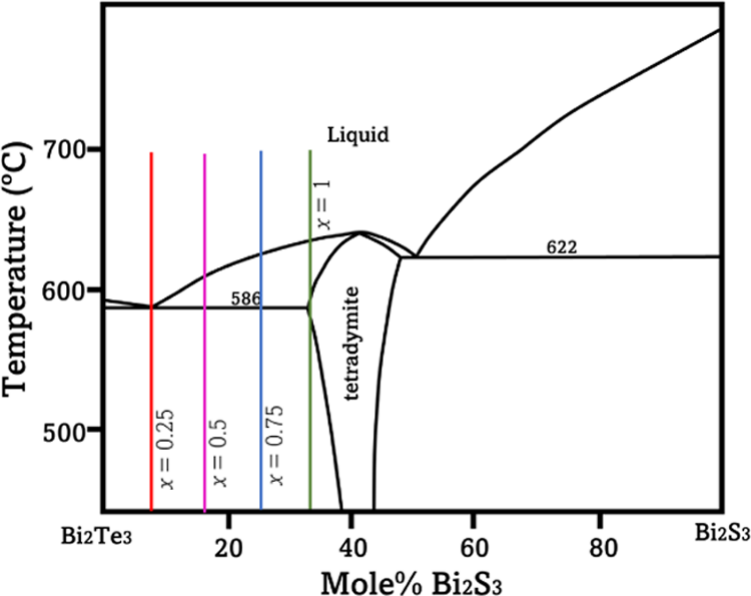
Phase diagram of Bi_2_Te_3_–Bi_2_S_3_, adapted from ref (25), indicating the composition of samples Bi_2_Te_3–*x*_S_*x*_ (*x* = 0, 0.25, 0.5, 0.75, and 1).

According to the pseudobinary phase diagram of Bi_2_Te_3_–Bi_2_S_3_^25^ (Figure 1), Bi_2_Te_3–*x*_S_*x*_ (*x* = 0.25, 0.5, and 0.75) compounds comprise two phases, namely, Bi_2_Te_3_ and Bi_2_Te_2_S.

**Figure 2:**
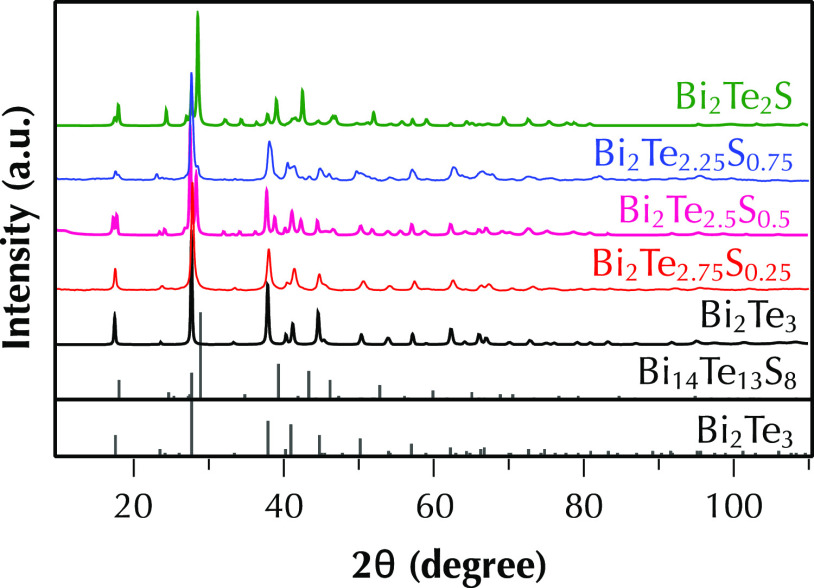
Room-temperature X-ray diffraction patterns of Bi_2_Te_3–*x*_S_*x*_ (*x* = 0, 0.25, 0.5, 0.75, and 1).

The purity and crystal structure of the undoped Bi_2_Te_3–*x*_S_*x*_ (*x* = 0, 0.25, 0.5, 0.75, and 1) samples are determined by indexing the powder X-ray diffraction (XRD) patterns, shown in Figure 2. All samples exhibit trigonal crystal structures. The Bi_2_Te_3_ sample is a single phase belonging to the *R*3̅*m* space group with the lattice parameters *a* = *b* = 4.39 Å, *c* = 30.47 Å, α = β = 90°, and γ = 120°. However, the sample stoichiometrically referred to as Bi_2_Te_2_S comprises two phases, namely, Bi_2_Te_3_ and Bi_14_Te_13_S_8_. The latter phase belongs to the *R*3̅ space group with the lattice parameters *a* = *b* = 4.18 Å, *c* = 29.45 Å, α = β = 90°, and γ = 120°. The tetradymites Bi_2_Te_3_ are based on five layers in three blocks in a [Te_2_–Bi–Te_1_–Bi–Te_2_ ]_0_ – [Te_2_–Bi–Te_1_–Bi–Te_2_]_1/3_ – [Te_2_–Bi–Te_1_–Bi–Te_2_]_2/3_ sequence with the subscripts indicating the *z* translation of the blocks within the hexagonal unit cell.^26^

In the case of Bi_2_Te_2_S, it was initially proposed that the S atoms substituted at Te sites.^27^ However, this structure was unstable at that composition due to the high strains on the internal sulfur layer caused by the electronegative anion of sulfur and the very different ionic radii of Te and S, resulting in a large size mismatch of the hexagonal-close-packed Te and S layers. Therefore, a more complex formula, Bi_14_Te_13_S_8_, was suggested for the single-phase structure where S only occupies the Te_1_ sites in the Bi_2_Te_3_ structure.^26,28^ This phase has an orthorhombic crystal structure, belongs to the *R*3̅ space group with lattice parameters of *a* = *b* = 11.24 Å and α = β = γ = 56.83° or a corresponding hexagonal structure with lattice parameters of *a* = 4.25 Å, *c* = 29.6 Å, α = β = 90°, and γ = 120°.^28^ The stoichiometrically prepared sample of Bi_2_Te_2_S contains less sulfur than single-phase Bi_14_Te_13_S_8_, and therefore, as XRD results indicate, it contains a secondary Bi_2_Te_3_ phase within the Bi_14_Te_13_S_8_ matrix.

It is worth noting that this crystal structure has been indexed as Bi_2_Te_2_S in the XRD databases of the Inorganic Crystal Structure Database (ICSD) and Open Quantum Materials Database (OQMD). Consequently, we used this to identify phases in our previous work,^24^ and in fact, the phase labeled the Bi_2_Te_2_S phase in our previous report^24^ is the Bi_14_Te_13_S_8_ phase. We acknowledge the incorrect labeling of the Bi_14_Te_13_S_8_ phase as Bi_2_Te_2_S in our previous study and refer to it as Bi_14_Te_13_S_8_ in this work. Rietveld refinement was also employed via the FullProf software suite to determine the proportion of phases, summarized in Table 1.

**Table 1:** Estimated Mass Ratio of the Phases in the Bi_2_Te_3–*x*_S_*x*_ (*x* = 0, 0.25, 0.5, 0.75, and 1) Samples Obtained by Rietveld Refinement

*x*	0	0.25	0.5	0.75	1
Bi_2_Te_3_	100	83.9 ± 1.5	55.4 ± 0.7	33.4 ± 1.8	18.9 ± 0.3
Bi_14_Te_13_S_8_		16.1 ± 0.8	44.6 ± 0.5	66.6 ± 0.7	81.1 ± 0.8

The electronic transport properties of bismuth chalcogenides are affected by the fraction of individual phases.^7,29^ Therefore, we employed a combination of an EBSD technique and EDS mapping to discriminate the Bi_2_Te_3_ and Bi_14_Te_13_S_8_ phases.^24^

**Figure 3:**
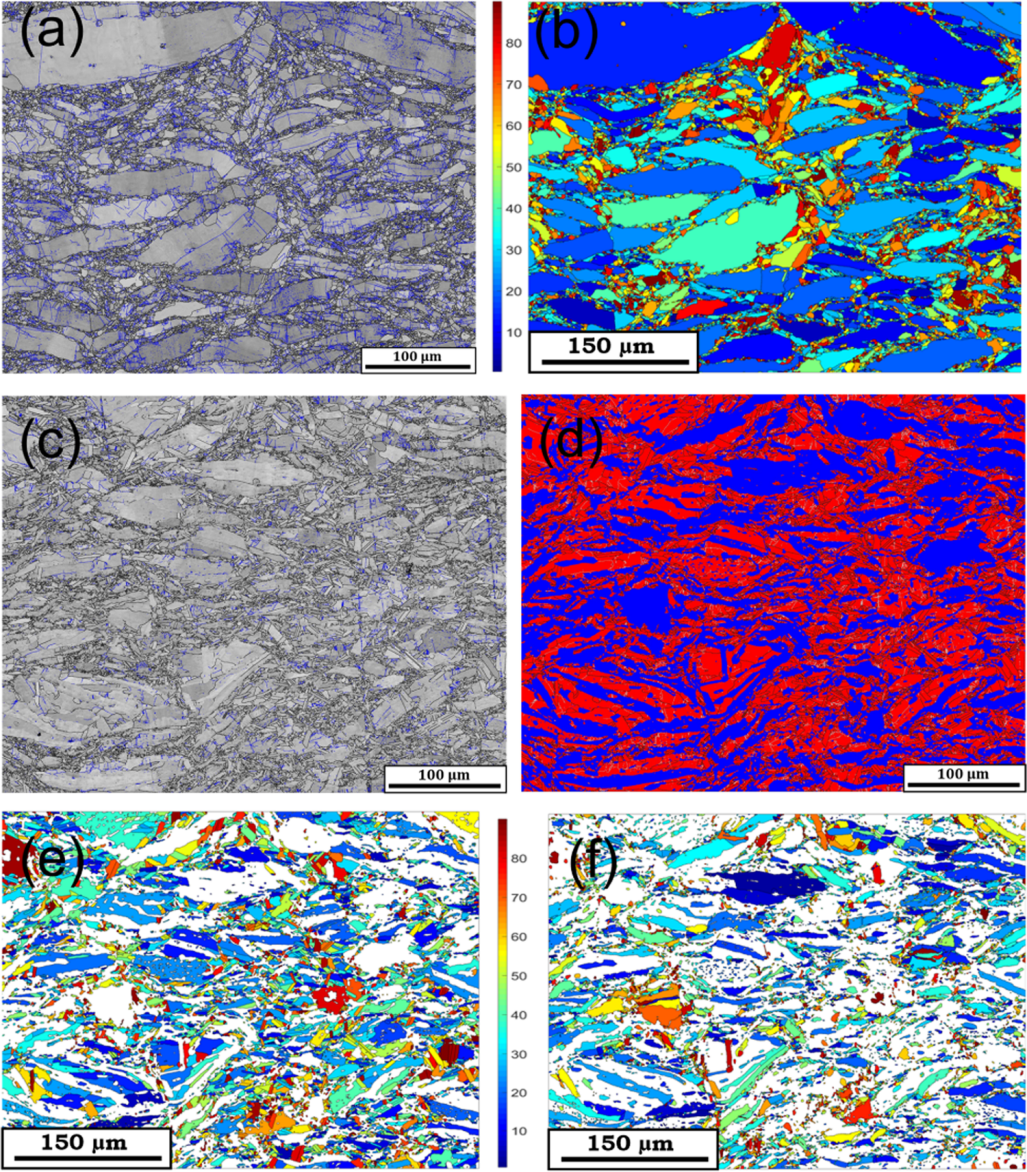
(a) Band contrast and (b) EBSD image of the Bi_2_Te_3_ sample, indicating a single-phase compound with grains elongated normal to the direction of sintering. (c) Band contrast, (d) phase distribution map of Bi_2_Te_3_ (red) and Bi_14_Te_13_S_8_ (blue), (e) EBSD image of the Bi_2_Te_3_ phase, and (f) EBSD image of the Bi_14_Te_13_S_8_ phase in the Bi_2_Te_2.5_S_0.5_ sample, showing the sample containing both phases with grains sizes much smaller than the single-phase Bi_2_Te_3_ sample. EBSD images indicate the orientation of the unit cells’ c-axis of each phase relative to the compression axis.

Figure 3a,c shows the band contrast maps of the Bi_2_Te_3–*x*_S_*x*_ (*x* = 0 and 0.5) sample cross section, the surface parallel to the sintering direction, respectively. The low-angle grain boundaries (LAGBs) are defined as misorientations between 2 ≤ θ ≤ 15° in blue and high-angle grain boundaries (HAGBs) as misorientations >15° in black. Since the powder was hand-ground from an annealed ingot in an agate mortar and pestle, it resulted in a large variation in grain sizes, with some grains exceeding 100 μm. In general, most large grains are elongated perpendicular to the direction of sintering. The latter is along the map vertically.

Figure 3d shows the phase distribution maps of Bi_2_Te_3_ (red) and Bi_14_Te_13_S_8_ (blue) phases in the multiphase sample of Bi_2_Te_2.5_S_0.5_. Although the same fabrication method was employed to prepare these samples, the grain sizes in the multiphase Bi_2_Te_2.5_S_0.5_ sample are much smaller than in single-phase Bi_2_Te_3_ (compare Figure 3a,c). The phase diagram of Bi_2_Te_3_–Bi_2_S_3_^25^ (Figure 1) shows that there is a eutectic transformation between Bi_2_Te_3_ and Bi_14_Te_13_S_8_, following which a lamellar structure is expected in the final ingot. This has resulted in much finer grain size in the multiphase sample of Bi_2_Te_2.5_S_0.5_ than in the single-phase Bi_2_Te_3_ sample.

Figure 3b shows the EBSD image of the Bi_2_Te_3_ phase in a single-phase sample of Bi_2_Te_3_, and Figure 3e,f shows the EBSD image of Bi_2_Te_3_ and Bi_14_Te_13_S_8_ phases, respectively, in the multiphase sample of Bi_2_Te_2.5_S_0.5_. These images show the orientation of the unit cells’ *c*-axis of each phase relative to the compression axis. The grains sizes of both phases in the Bi_2_Te_2.5_S_0.5_ sample are very similar and elongated perpendicular to the sintering direction, similar to the single-phase sample.

### 3.2 Electronic Transport Properties of Bi_2_Te_3–*x*_S_*x*_ (*x* = 0, 0.25, 0.5, 0.75, and 1) Samples

**Figure 4:**
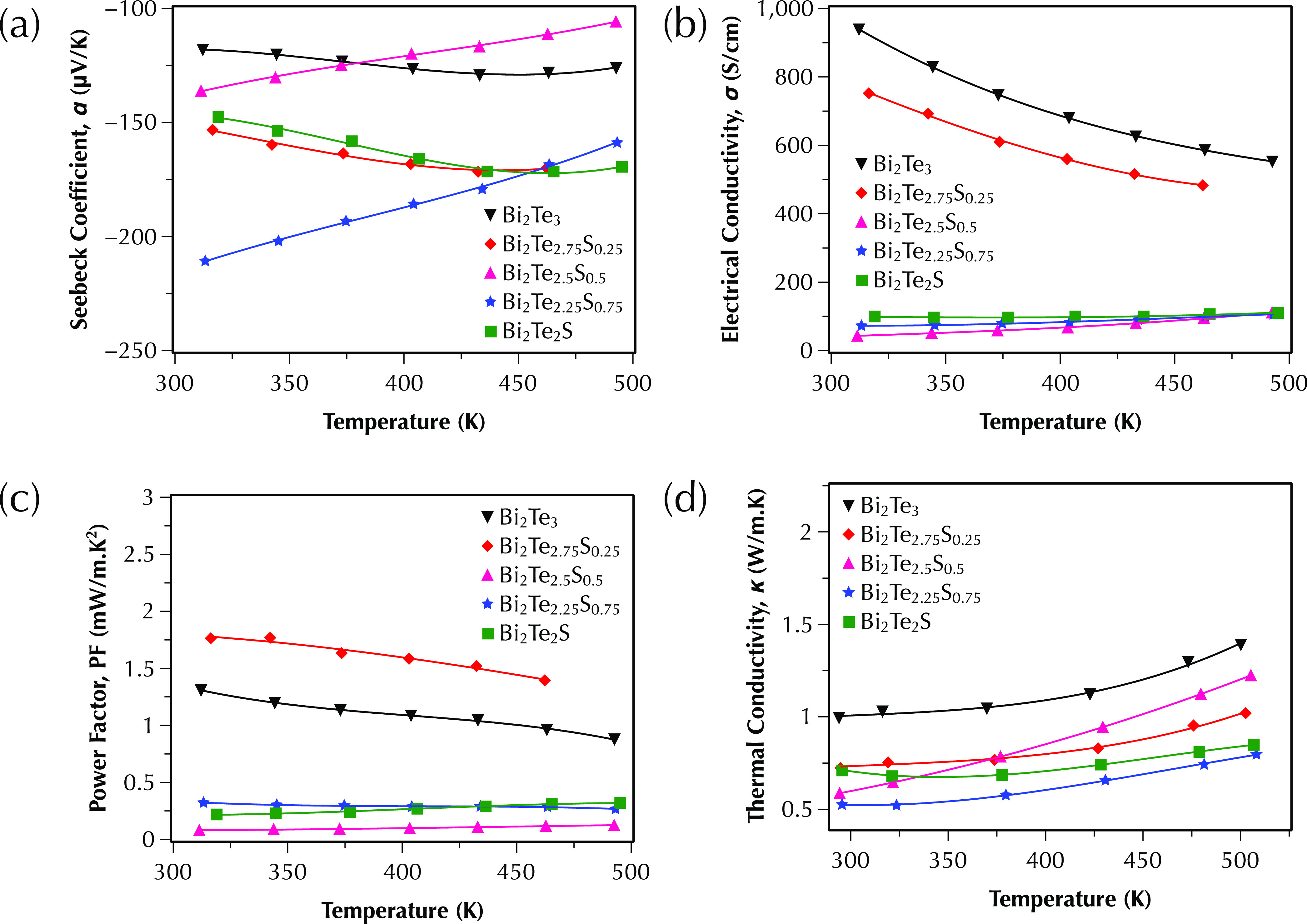
Temperature dependence between 300 and 500 K of (a) the Seebeck coefficient, (b) the electrical conductivity, (c) the power factor, and (d) the total thermal conductivity of Bi_2_Te_3–*x*_S_*x*_ (*x* = 0, 0.25, 0.5, 0.75, and 1) samples parallel to the sintering direction.

Figure 4 shows the temperature-dependent thermoelectric properties of the undoped Bi_2_Te_3–*x*_S_*x*_ (*x* = 0, 0.25, 0.5, 0.75, and 1) samples between 300 K and 500 K. All thermoelectric properties of this set of samples were measured parallel to the sintering direction.

All samples show negative Seebeck coefficient values, which indicates that electrons compose most charge carriers. The Seebeck coefficient (Figure 4a) of all samples with *x* = 0.5 and 0.75 decreases with temperature, exhibiting a temperature dependence behavior typical of intrinsic semiconductors. The Bi_2_Te_2_S and Bi_2_Te_3_ samples show a bipolar effect within the range of 400–500 K that is typical behavior of narrow band gap degenerate semiconductors^30^ where the intrinsic carriers are excited and compete with the extrinsic carriers, which in turn decreases the Seebeck coefficient with increasing the temperature as a result of the combined contribution of negative and positive charge carriers.

The electrical conductivity (Figure 4b) of all samples, except for samples with *x* = 0 and 0.25, increases with temperature and exhibits a temperature dependence behavior, typical of intrinsic semiconductors. The highest value of power factors, ∼1.75 mW/m·K^2^ (Figure 4c) was obtained in the multiphase Bi_2_Te_2.75_S_0.25_ sample (containing roughly 16 wt % of Bi_14_Te_13_S_8_ in the matrix of Bi_2_Te_3_) at around room temperature with a Seebeck coefficient of ∼150 μV/K and above 1.5 mW/m·K^2^ over the whole temperature range. This marks a significant increase from ∼1.25 mW/m·K^2^ for the single-phase Bi_2_Te_3_.

**Figure 5:**
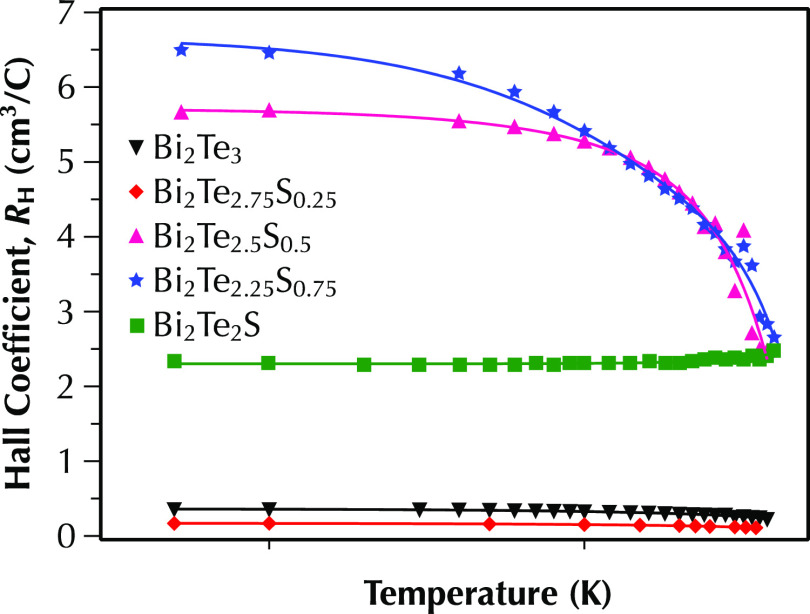
Temperature dependence of the Hall coefficient, *R*_H_, for Bi_2_Te_3–*x*_S_*x*_ (*x* = 0, 0.25, 0.5, 0.75, and 1) samples between 5 and 400 K.

The temperature-dependent Hall coefficient, *R*_H_, of Bi_2_Te_3–*x*_S_*x*_ (*x* = 0, 0.25, 0.5, 0.75, and 1) samples between 10 and 400 K (Figure 5) shows that the Hall coefficient of multiphase samples with *x* = 0.5 and 0.75 decreases with temperature, while the values are constant over the whole temperature range for the rest of samples. The Hall carrier concentrations (*n*_H_) of all samples were calculated at 350 K and are summarized in Table 2. These samples are all undoped and the charge carriers are caused by the structural defects. The multiphase samples of Bi_2_Te_3–*x*_S_*x*_ (*x* = 0.5, 0.75, and 1) with a larger fraction of the Bi_14_Te_13_S_8_ secondary phase show very low charge carrier concentrations (∼10^18^ cm^–3^), while the carrier concentration of the multiphase sample of Bi_2_Te_2.75_S_0.25_ is ∼1.2 × 10^19^, similar to the charge carrier concentration of Bi_2_Te_3_ (∼1 × 10^19^), explaining the higher electrical conductivity and the behavior, typical of a degenerate semiconductor. The optimum thermoelectric performance of n-type Bi_2_Te_3_ is usually achieved at carrier concentrations in the lower range of ×10^19^ cm^–3^,^31^ similar to the single-phase sample of Bi_2_Te_3_ and the multiphase sample of Bi_2_Te_2.75_S_0.25_. All samples of this study are undoped, and these results suggest that the variations in the charge carrier concentrations in these materials might not be solely due to a substitutional effect of Te with S in Bi_2_Te_3_ but likely due to the random formation of antisites and vacancy defects and various ratios of each phase in these samples. Table 2 summarizes the Seebeck coefficient, carrier concentration, and mobility of Bi_2_Te_3–*x*_S_*x*_ (*x* = 0, 0.25, 0.5, 0.75, and 1) samples at room temperature.

**Table 2:** Room-Temperature Resistivity (ρ), Seebeck Coefficient (α), Hall Charge Carrier Concentration (*n*_H_), and Hall Mobility (μ_H_) of Bi_2_Te_3–*x*_S_*x*_ (*x* = 0, 0.25, 0.5, 0.75, and 1) Samples

*x*	resistivity, ρ (mΩ·cm)	Seebeck coefficient, α (μV/K)	Hall carrier concentration, *n*_H_ (cm^–3^)	Hall mobility, μ_H_ (cm^2^/V·s)
0	0.94	–118	1.0 × 10^19^	655
0.25	1.80	–153	1.2 × 10^19^	284
0.5	17.9	–136	1.9 × 10^18^	183
0.75	17.2	–210	1.7 × 10^18^	213
1	14.3	–147	2.7 × 10^18^	161

**Figure 6:**
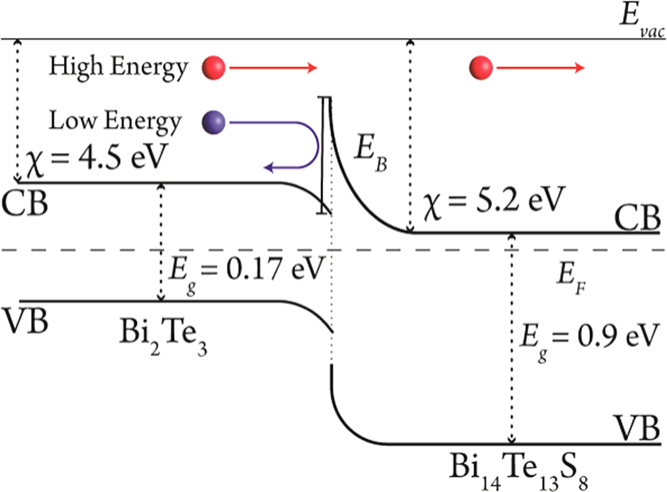
Schematic diagram of the energy filtering effect at the interface between Bi_2_Te_3_ and Bi_14_Te_13_S_8_.

Although the point defects have controlled the carrier concentration of samples and effects on their electrical conductivity significantly, regardless of the carrier concentrations, all multiphase samples of Bi_14_Te_13_S_8_–Bi_2_Te_3_ (Bi_2_Te_3–*x*_S_*x*_ (*x* = 0.25, 0.5, 0.75, and 1)) show much higher Seebeck coefficients than the single-phase Bi_2_Te_3_. The Bi_2_Te_2.75_S_0.25_ sample with carrier concentration similar to Bi_2_Te_3_ (∼1.0 × 10^19^) have shown the Seebeck coefficient of ∼150 μV/K, much higher than the value of ∼120 μV/K for single-phase Bi_2_Te_3_ at room temperature (Figure 4a). This can be explained by the energy filtering effect, where the potential barriers generated in the electronic band structure of the matrix through interfaces with the secondary phases increases the overall Seebeck coefficient.^13,18^ In this effect, low energy carriers are scattered by potential barriers formed at the junction of the two phases. The barrier height can be approximated as a function of the difference between the electron affinity of two phases. The multiphase samples of Bi_14_Te_13_S_8_–Bi_2_Te_3_ (Bi_2_Te_3–*x*_S_*x*_ (*x* = 0.25, 0.5, 0.75, and 1)) have an estimated barrier height of ≈ 0.7 eV, considering the electron affinity, χ, of ≈5.26 eV for Bi_14_Te_13_S_8_^32^ and ≈4.5 eV for Bi_2_Te_3_,^33^ schematically demonstrated in Figure 6.

The temperature dependence of the total thermal conductivity, κ, between 300 and 500 K for all Bi_2_Te_3–*x*_S_*x*_ (*x* = 0, 0.25, 0.5, 0.75, and 1) samples is presented in Figure 4d. The total thermal conductivity of all samples increases with temperature, indicating low carrier concentrations in these samples. In general. all multiphase samples of Bi_2_Te_3–*x*_S_*x*_ (*x* = 0.25, 0.5, 0.75, and 1) show the total thermal conductivities much lower than the single-phase Bi_2_Te_3_ sample. The total thermal conductivity of the highly conductive multiphase Bi_2_Te_2.75_S_0.25_ sample, with similar charge carrier concentration to Bi_2_Te_3_, is below ∼1.1 W/m·K over the temperature range as a result of the finer microstructure (Figure 3).

**Figure 7:**
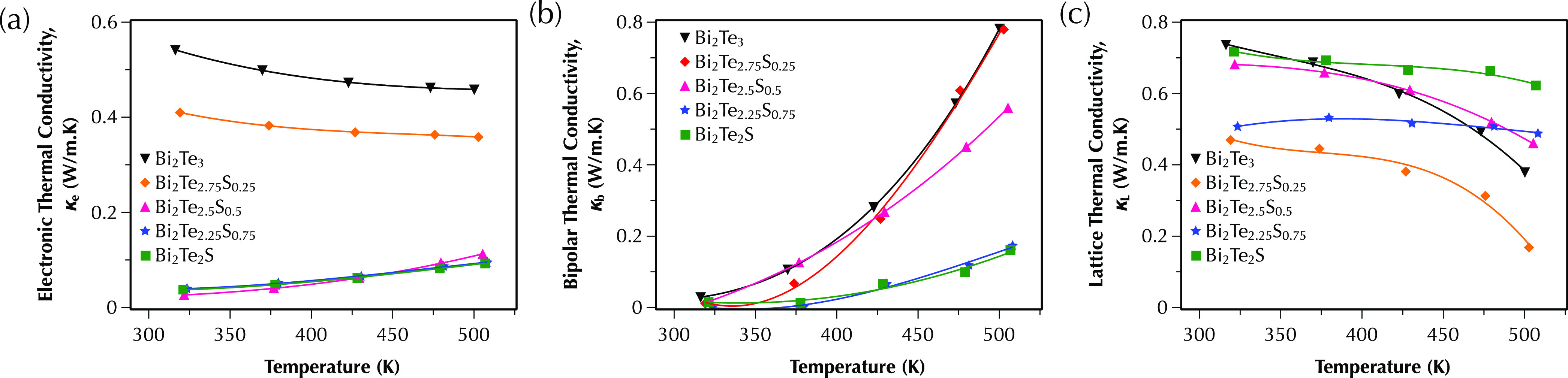
Temperature dependence of the (a) electronic thermal conductivity; (b) bipolar thermal conductivity; and (c) lattice thermal conductivity for Bi_2_Te_3–*x*_S_*x*_ (*x* = 0, 0.25, 0.5, 0.75, and 1) samples between 300 and 500 K measured parallel to the sintering direction.

**Figure 8:**
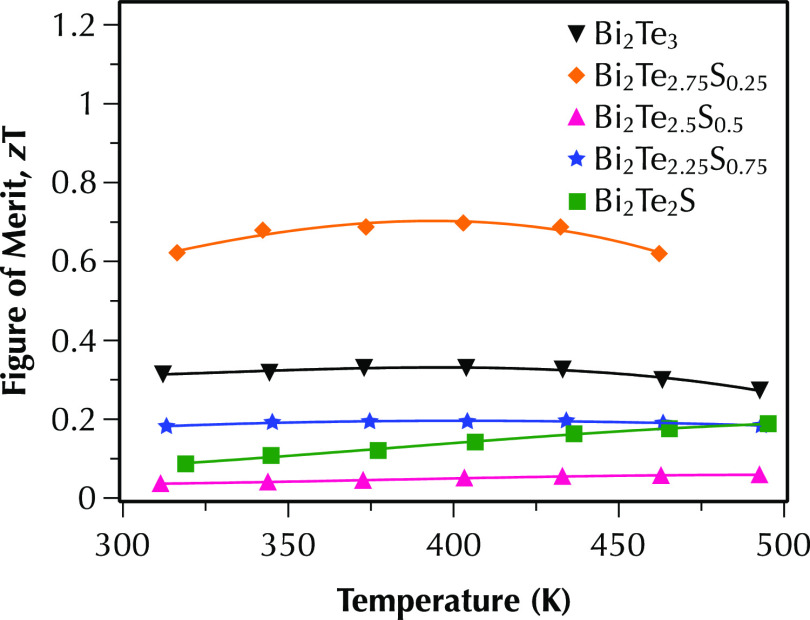
Temperature dependence of the figure of merit, *zT*, for Bi_2_Te_3–*x*_S_*x*_ (*x* = 0, 0.25, 0.5, 0.75, and 1) samples between 300 and 500 K measured parallel to the sintering direction.

To understand the effect of the microstructure on the lattice thermal conductivity of these samples, a multiband parabolic model with one electron (n) band and one hole (p) band was employed to calculate the electronic and bipolar contributions of the thermal conductivity for all samples.^34^ The multiband model considers both holes and electrons, and the overall Seebeck coefficient and electrical conductivity of samples can be described by
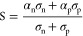
1
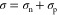
2where α_n_, α_p_, σ_n_, and σ_p_ are the partial Seebeck coefficient and electrical conductivity of electrons and holes, respectively. The electronic (κ_e_) and bipolar (κ_b_) contributions of the thermal conductivity are given as
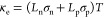
3
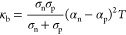
4where *L*_n_ and *L*_p_ are the Lorenz numbers of the conduction and valence bands, respectively. More details of the calculations are provided in the Supporting Information. The electronic (κ_e_), bipolar (κ_b_), and lattice (κ_*L*_ = κ – κ_e_ – κ_b_) thermal conductivities of Bi_2_Te_3–*x*_S_*x*_ (*x* = 0, 0.25, 0.5, 0.75, and 1) samples are presented in Figure 7. The most electrically conductive samples (*x* = 0 and 0.25) show higher values of electronic contribution to the total lattice thermal conductivities than the less conductive ones (Figure 7a). The bipolar thermal conductivity of all samples increases with temperature and decreases with an increase in the fraction of the secondary phase (Figure 7b) due to the larger band gap of Bi_14_Te_13_S_8_. The lattice thermal conductivity of all samples decreases with the temperature, indicating the phonon scattering mechanisms.^35^ All multiphase samples with a fine microstructure (*x* = 0.25, 0.5, and 0.75) show lower lattice thermal conductivity than Bi_2_Te_3_, most likely due to additional scattering of phonons at the defects, the grain boundaries, and the interfaces between two phases. The bipolar and lattice thermal conductivities of samples are calculated according to the multiband model. This model is developed for single-phase compounds, whereas there is a significant fraction of secondary phase existing in the samples of the current study; this introduces substantial errors in the calculated values of bipolar and lattice thermal conductivities of multiphase samples. Overall, the combination of low total thermal conductivity and high power factor in the highly conductive multiphase Bi_2_Te_2.75_S_0.25_ sample results in a reasonably high figure of merit of ∼0.7 (Figure 8) with values higher than 0.6 over the full temperature range of 300–500 K.

The multiphase samples of Bi_2_Te_2.75_S_0.25_, containing Bi_14_Te_13_S_8_ and Bi_2_Te_3_, were doped with iodine to obtain samples of  (*y* = 0.005, 0.01, and 0.02), with various charge carrier concentrations. The thermoelectric performance of these samples was measured for both directions parallel and perpendicular to the sintering orientation.

### 3.3 Electronic Transport Properties of (y = 0.005, 0.01, and 0.02) Samples

**Figure 9:**
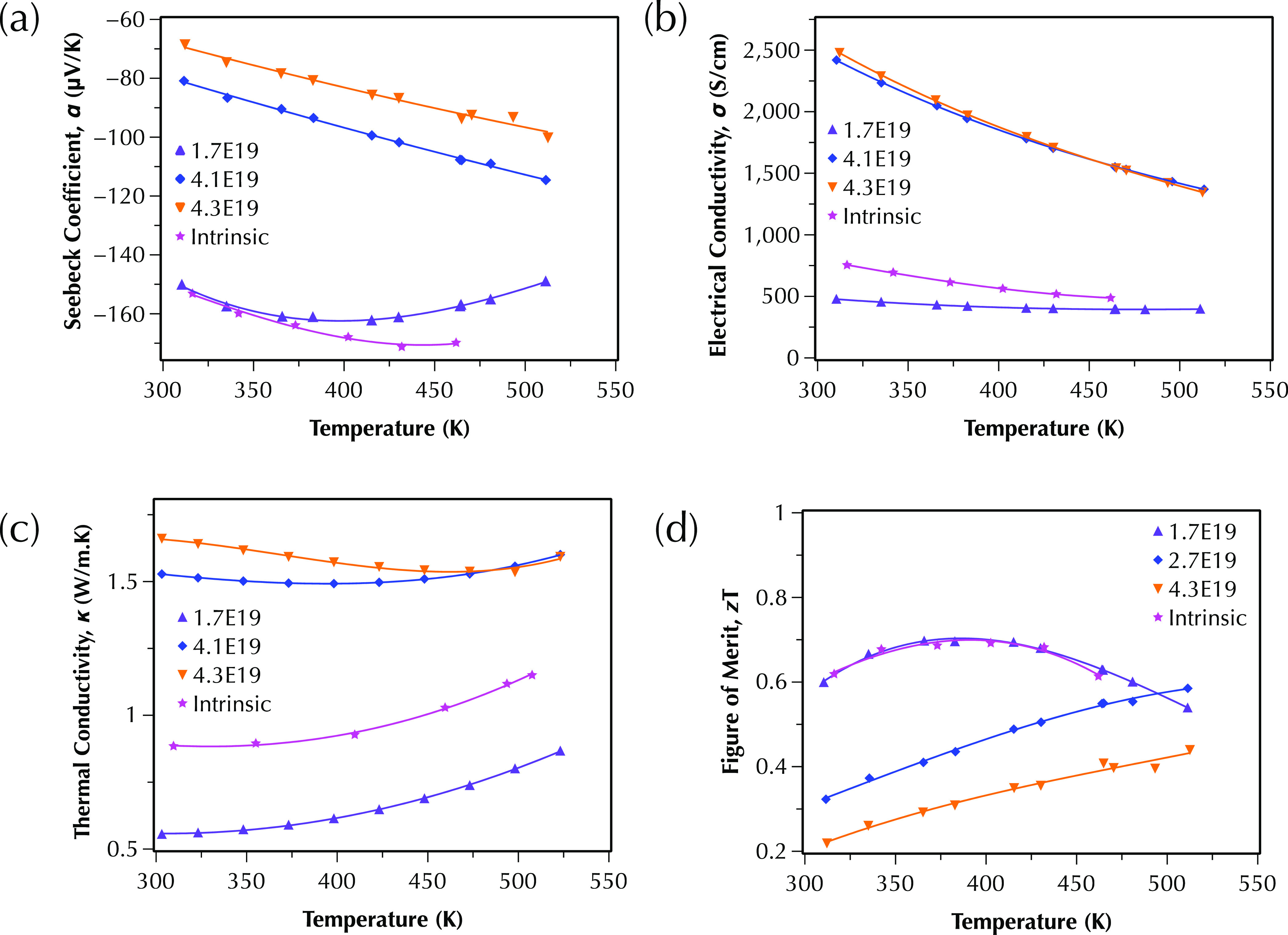
Temperature dependence of (a) the Seebeck coefficient; (b) the electrical conductivity; (c) the total lattice thermal conductivity; and (d) the figure of merit, *zT*, of  (*y* = 0.005, 0.01, and 0.02) samples between 300 and 500 K measured parallel to the sintering direction, compared with the intrinsic sample shown in Figure 4.

The temperature-dependent Seebeck coefficient, electrical conductivity, total thermal conductivity, and figure of merit of iodine-doped multiphase Bi_2_Te_2.75_S_0.25_ compound, measured parallel to the sintering direction at the temperature range of 300–530 K, are shown in Figure 9a–d, respectively. The thermoelectric performance of these samples was also measured at the direction perpendicular to the sintering direction (detailed in the Supporting Information). The electronic transport properties of these samples are compared with the intrinsic sample, shown in Figure 4. The samples are named with their Hall carrier concentrations in Figure 9 for simplicity of comparison. The electrical conductivity has been increased and the Seebeck coefficient has been reduced by the increase in the carrier concentration, as expected; the intrinsic sample has shown only slightly lower charge carrier concentrations (1.2 × 10^19^ cm^–3^) than the slightly doped sample (1.7 × 10^19^ cm^–3^); however, it has higher electrical conductivity and higher thermal conductivity than the slightly doped samples. We have used the same fabrication method to prepare doped samples as the intrinsic ones; however, the SPS equipment used to sinter these samples was changed, which had lower accuracy in the pressure control system at lower ranges, resulting in samples with densities (ρ) more than 90% of the relative densities, much lower than the density of intrinsic samples (>99% of the theoretical density) that were prepared by different equipment. The porosities in the doped samples resulted in lower electrical conductivity of samples with the same chemistry and carrier concentrations due to scattering of electrons.^36^ However, it also reduced the thermal conductivity (Figure 9c) of samples due to the scattering of phonons in the porous structure. This resulted in a similar figure of merit for both samples, showing that optimum charge carrier concentrations for this composition is also in the lower range of ×10^19^ cm^–3^, similar to the single-phase n-type Bi_2_Te_3_ sample.^31^ The intrinsic Bi_2_Te_3_ sample prepared in this study (Table 2 and Figure 4) showed the charge carrier concentrations at the lower range of ×10^19^ cm^–3^, proving a reasonable comparison of the thermoelectric performance of multiphase samples with the single-phase n-type Bi_2_Te_3_.

These results suggest that there is a possibility to obtain high room temperature and/or over a wide temperature range thermoelectric performance in the multiphase pseudobinary Bi_14_Te_13_S_8_–Bi_2_Te_3_ alloys. These samples exhibit a high power factor and simultaneously provide low total thermal conductivities.

## 4 Conclusions

In summary, multiphase pseudobinary Bi_14_Te_13_S_8_–Bi_2_Te_3_ compounds are promising high-performance thermoelectric materials around room temperature. We have shown that the finely distributed secondary phase of Bi_14_Te_13_S_8_ with a larger band gap and electron affinity than the matrix Bi_2_Te_3_ increased the Seebeck coefficient through the energy filtering effect. This resulted in a higher power factor of multiphase compounds relative to single-phase Bi_2_Te_3_. Simultaneously, the increased number of interfaces between the two phases formed through eutectic transformation during solidification of the compounds added additional scattering centers for phonons, leading to a decrease in the lattice thermal conductivity of multiphase samples. The combination of these beneficial effects resulted in a reasonably high figure of merit of ∼0.7 over the temperature range of 300–550 K for the multiphase sample of Bi_2_Te_2.75_S_0.25_, doubling the efficiency of single-phase Bi_2_Te_3_.

## Supporting Information Available

More detailed information including XRD results, heat capacities, electronic transport properties of samples perpendicular to the direction of sintering, and detailed multiband model used to define bipolar and lattice thermal conductivities of samples (PDF)
