# Supplementary material for: Room-Temperature Thermoelectric Performance of n-Type Multiphase Pseudobinary Bi2Te3–Bi2S3 Compounds: Synergic Effects of Phonon Scattering and Energy Filtering
Source: ACS Appl Mater Interfaces. 2023 Apr 4;15(15):19220–9. doi: 10.1021/acsami.3c01956 (PMC10119860; doi:10.1021/acsami.3c01956)
Supplement: Supplementary file 1 — am3c01956_si_001.pdf [file am3c01956_si_001.pdf]

Supporting Information for

Room Temperature Thermoelectric Performance of *n*-type Multiphase Pseudo-Binary Bi_2Te_3 – Bi_2S_3 Compounds: Synergic Effects of Phonon Scattering and Energy Filtering

Sima Aminorroaya Yamini[1, 2], Rafael Santos[3], Raphael Fortulan[2], Azdiar A Gazder[3], Abhishek Malhotra[4], Daryoosh Vashaee[4], Illia Serhiienko[5], Takao Mori[5,6]*

[1] Department of Engineering and Mathematics, Sheffield Hallam University, Sheffield S1 1 WB, UK.

[2] Materials and Engineering Research Institute, Sheffield Hallam University, Sheffield S1 1WB, UK.

[3] Australian Institute for Innovative Materials (AIIM), University of Wollongong, New South Wales, 2500, Australia.

[4] Department of Materials Science and Engineering, North Carolina State University, Raleigh, Raleigh, North Carolina, 27606, USA.

[5] International Centre for Materials Nanoarchitectonics (WPI-MANA), National Institute for Materials Science, Tsukuba, 305-0044, Japan.

[6] Graduate School of Pure and Applied Science, University of Tsukuba, Tsukuba, 305-8577, Japan.

* Email: S.Aminorroaya@shu.ac.uk

XRD Analysis

The quantitative phase composition obtained of using the Rietveld refinement of the PXRD patterns for $Bi_2Te_{3-x}S_x$ (x = 0, 0.25, 0.5, 0.75 and 1) samples shown in Figure S1.

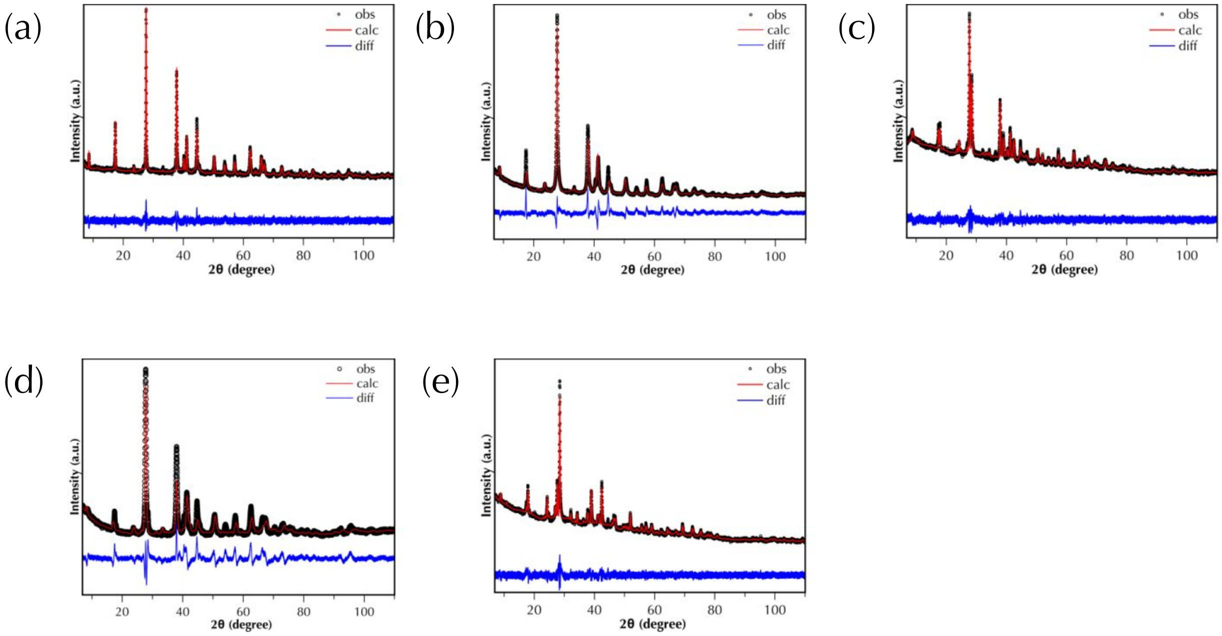

Figure S1. (a) to (e): Rietveld refinement plots of $Bi_2Te_{3-x}S_x$ (x = 0, 0.25, 0.5, 0.75, and 1) samples, respectively.

Table S1. Rietveld refined lattice parameters of $Bi_2Te_{3-x}S_x$ (x = 0, 0.25, 0.5, 0.75, and 1) samples,

	**Bi_2Te_3**		**$Bi_{14}Te_{13}S_8$**	
**Composition**	a = b [nm]	c [nm]	a = b [nm]	c [nm]
Bi_2Te_3	0.438239(15)	3.04809(8)	—	—
$Bi_2Te_{2.75}S_{0.25}$	0.439718(7)	3.046549(5)	1.124201(21)	2.961038(5)
$Bi_2Te_{2.5}S_{0.5}$	0.437239(16)	2.9698(7)	1.12496(5)	2.9610(2)
$Bi_2Te_{2.5}S_{0.75}$	0.4395(4)	3.03993(12)	1.12494(5)	2.9622(2)
Bi_2Te_2S	0.43815(3)	3.0486(4)	1.12489(3)	2.95988(13)

Figure S2 shows the diffraction patterns of the $Bi_2Te_{2.75(1-\frac{y}{3})}S_{0.25(1-\frac{y}{3})}I_y$ (y = 0.005, 0.01, and 0.02) samples. All patterns confirm the presence of primary phase of trigonal Bi_2Te_3 belonging to the $R\bar{3}m$ space group and a secondary phase of orthorhombic $Bi_{14}Te_{13}S_8$ belongs to $R\bar{3}$ space group.

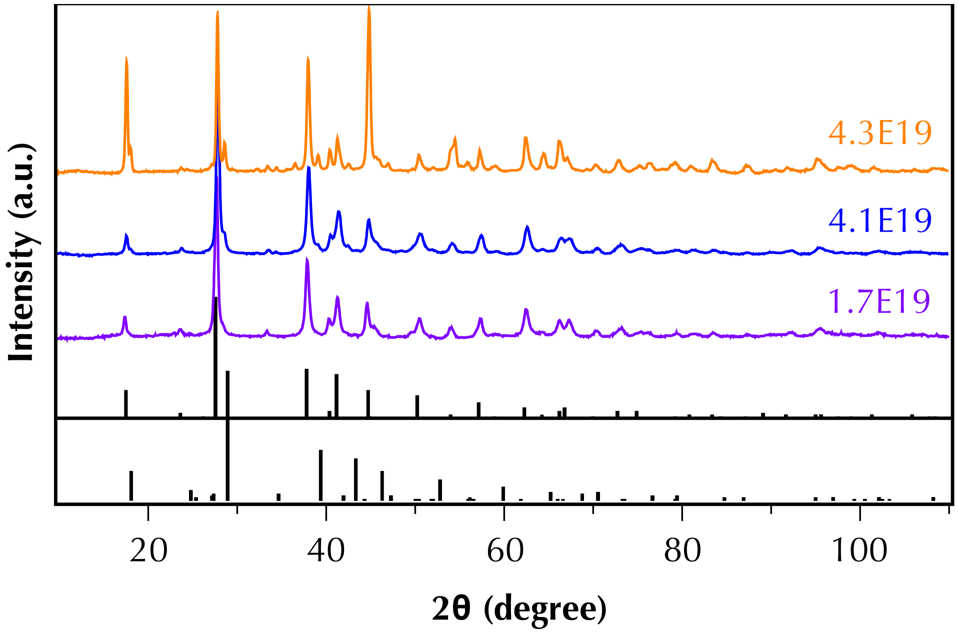

Figure S2. Room temperature X-ray diffraction patterns of $Bi_2Te_{2.75(1-\frac{y}{3})}S_{0.25(1-\frac{y}{3})}I_y$ (y = 0.005, 0.01, and 0.02) samples

Heat Capacity of $Bi_2Te_{3-x}S_x$ (x = 0, 0.25, 0.5, 0.75, and 1) samples

In Figure S3 shows the heat capacity measurements using DSC for the single phase Bi_2Te_3 (black dots) and multiphase $Bi_2Te_{2.75}S_{0.25}$ (green rectangles) and compare with the $C_p = 0.177981 + 7.11824 \times 10^5 T$ equation (green dashed line) and the one measured with LFA (purple rectangles). The DSC measurements were made according to the ASTM standard [1] and the assumed measurement error is around 5%.

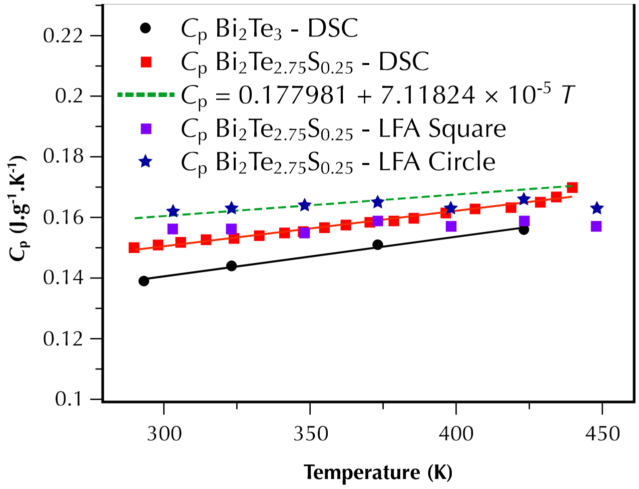

Figure S3. Comparison of DSC and LFA measurements of heat capacity from Bi_2Te_3, $Bi_2Te_{2.75}S_{0.25}$, and the calculated heat capacity of Bi_2Te_3 using $C_p = 0.177981 + 7.11824 \times 10^5 T$ as a function of temperature.

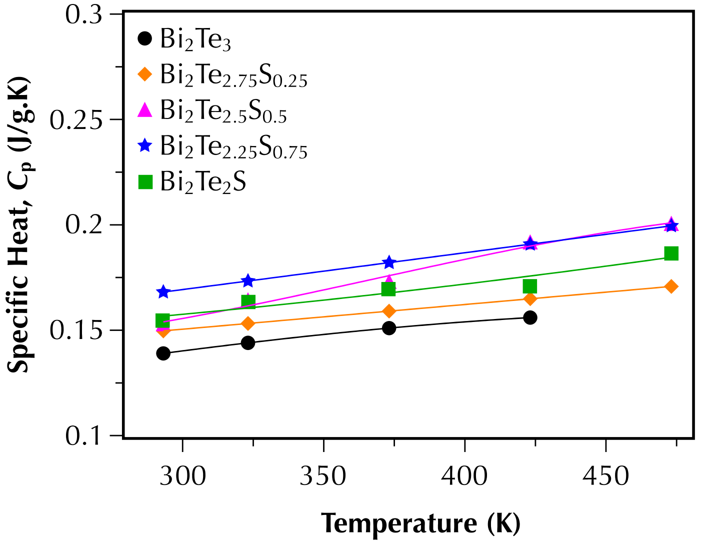

Figure S4. Heat capacity of $Bi_2Te_{3-x}S_x$ (x = 0, 0.25, 0.5, 0.75, and 1) samples, measured by the DSC.

Electronic transport properties of $Bi_2Te_{2.75(1-\frac{y}{3})}S_{0.25(1-\frac{y}{3})}I_y$ (y = 0.005, 0.01, and 0.02), samples perpendicular to the sintering direction

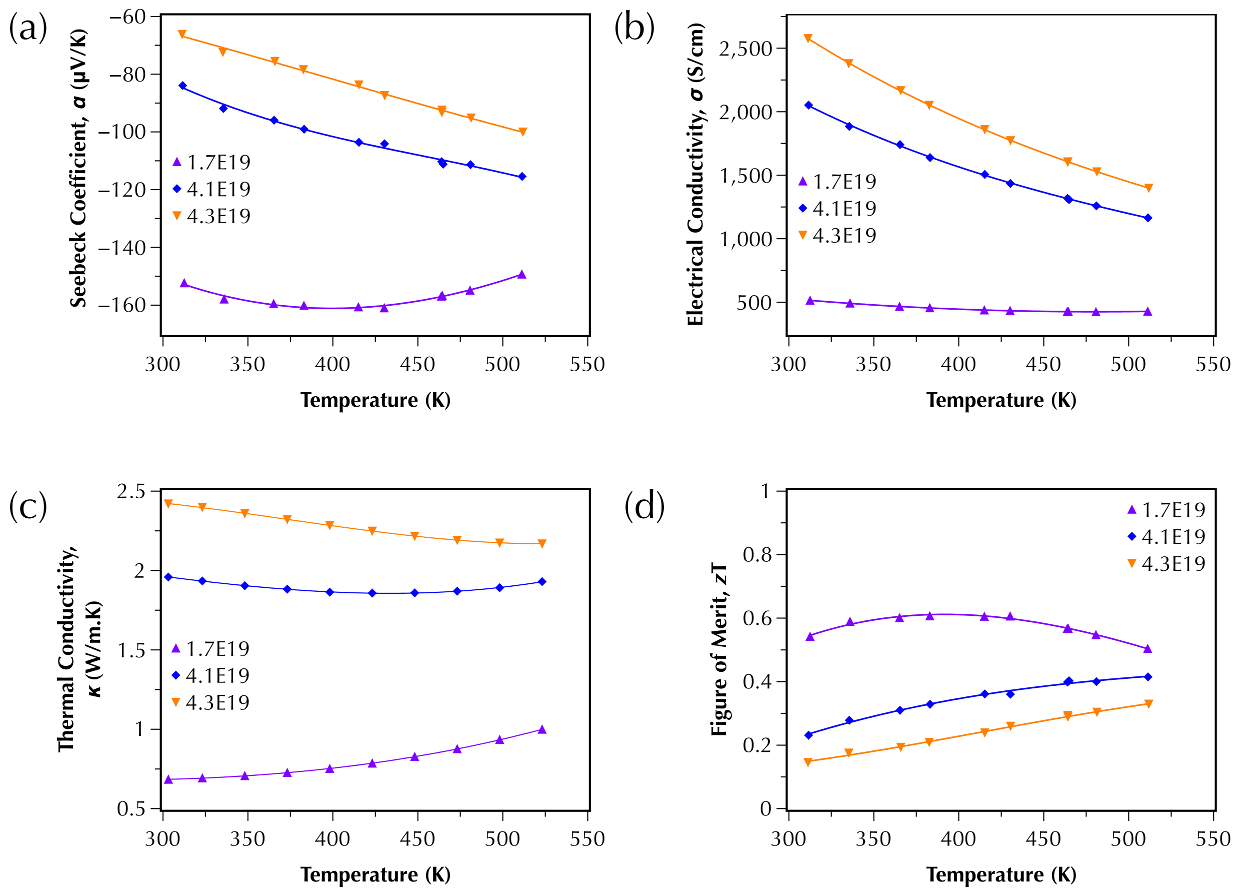

Figure S5. Temperature dependence of (a) the Seebeck coefficient; (b) the electrical conductivity; (c) total lattice thermal conductivity; and (d) the figure of merit, zT, of $Bi_2Te_{2.75(1-\frac{y}{3})}S_{0.25(1-\frac{y}{3})}I_y$ (y = 0.005, 0.01, and 0.02) samples between 300 and 500 K, perpendicular to the sintering direction.

The room temperature Seebeck coefficient as a function of carrier concentration is plotted for $Bi_2Te_{2.75(1-\frac{y}{3})}S_{0.25(1-\frac{y}{3})}I_y$ (y = 0.005, 0.01, and 0.02) samples in Figure S6. No bipolar effect was observed for these samples, therefore, a single parabolic band model with acoustic phonon scattering was assumed to evaluate the charge carrier effective mass using:

$$S = \frac{k_B}{q}\left(2\frac{F_1(\eta)}{F_0(\eta} - \eta\right),\#(1)$$

$$n = \frac{\left(2m^* k_B T\right)^{\frac{3}{2}}}{3\pi^2\hbar^3} F_{\frac{1}{2}}(\eta),\#(2)$$

where $\eta = E_F/(k_B T)$ is the reduced Fermi level, m^* is the effective mass, and $F_j(\eta)$ is Fermi integral given by:

$$F_j(\eta) = \int_0^\infty \frac{\epsilon}{1 + e^{\epsilon - \eta}} \mathrm{d}\epsilon\#(3)$$

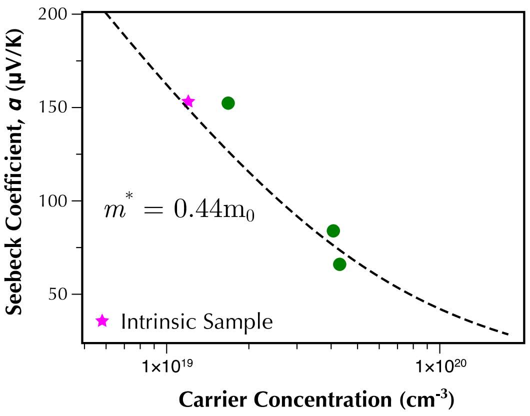

Figure S6. Room temperature Seebeck coefficient as a function of charge carrier concentration for $Bi_2Te_{2.75(1-\frac{y}{3})}S_{0.25(1-\frac{y}{3})}I_y$ (y = 0.005, 0.01, and 0.02) samples.

Electronic transport properties of $Bi_2Te_{3-z}S_z$ (z = 0, 0.5, 1, and 1.5) samples

A set of polycrystalline $Bi_2Te_{3-z}S_z$ (z = 0, 0.5, 1.0, 1.5) samples were synthesised by direct reaction of stoichiometric amounts of high purity Bi (99.999%, Alfa Aesar), Te (99.999%) shots and dried S (99.99%) powder in vacuum sealed quartz ampoules in an Ar glove box. The ampules were homogenised at 1123 K for 10 h, quenched in cold water, and annealed at 673 K for 72 hours. The obtained ingots were hand ground to fine powders in an agate mortar and pestle housed inside

an Ar atmosphere glove box. The powders were then loaded into a graphite die and sintered under vacuum to produce 12 mm diameter pellets using spark plasma sintering at 633 K and an axial pressure of 50 MPa for 5 min.

Figure S6 shows the temperature dependant thermoelectric properties of the undoped $Bi_2Te_{3-z}S_z$ (z = 0, 0.5, 1.0, 1.5) samples between 300 K and 500 K. The Seebeck coefficient and electrical conductivity of these samples were measured perpendicular to the sintering direction whereas the thermal conductivities were measured parallel to the sintering direction. Therefore, the figure of merit (zT) is not presented here to avoid overestimation of the thermoelectric efficiencies.

The electrical conductivity (Figure S7(a)) of all samples, except for the sample with z = 0.5, increases with temperature and exhibits a temperature dependence behaviour, typical of intrinsic semiconductors. The highest values of power factors, ~1.9 mW/m.K^2 (Figure S7(c)) was obtained in the multiphase $Bi_2Te_{2.5}S_{0.5}$ sample (containing roughly 50 wt% of $Bi_{14}Te_{13}S_8$ and Bi_2Te_3) at 350 K with a Seebeck coefficient of ~130 μV/K, and above 1.6 mW/m.K^2 over the whole temperature range. This marks a significant increase from ~1.2 mW/m.K^2 for the single phase Bi_2Te_3 and ~0.4-0.6 mW/m.K^2 for multiphase alloys of $Bi_2Te_{3-z}S_z$ (z = 1 and 1.5) with larger fractions of the $Bi_{14}Te_{13}S_8$ phase.

The temperature dependent Hall coefficient (R_H), of all samples perpendicular to the sintering direction are measured between 10 and 400 K (Figure S8). The Hall coefficient of single phase Bi_2Te_3 decreases with temperature while the values are constant over the temperature range for multiphase samples. The Hall carrier concentrations (n_H) of all samples were calculated at 350 K and summarised in Table S2. These samples are all undoped and the charge carriers are caused by the structural defects.

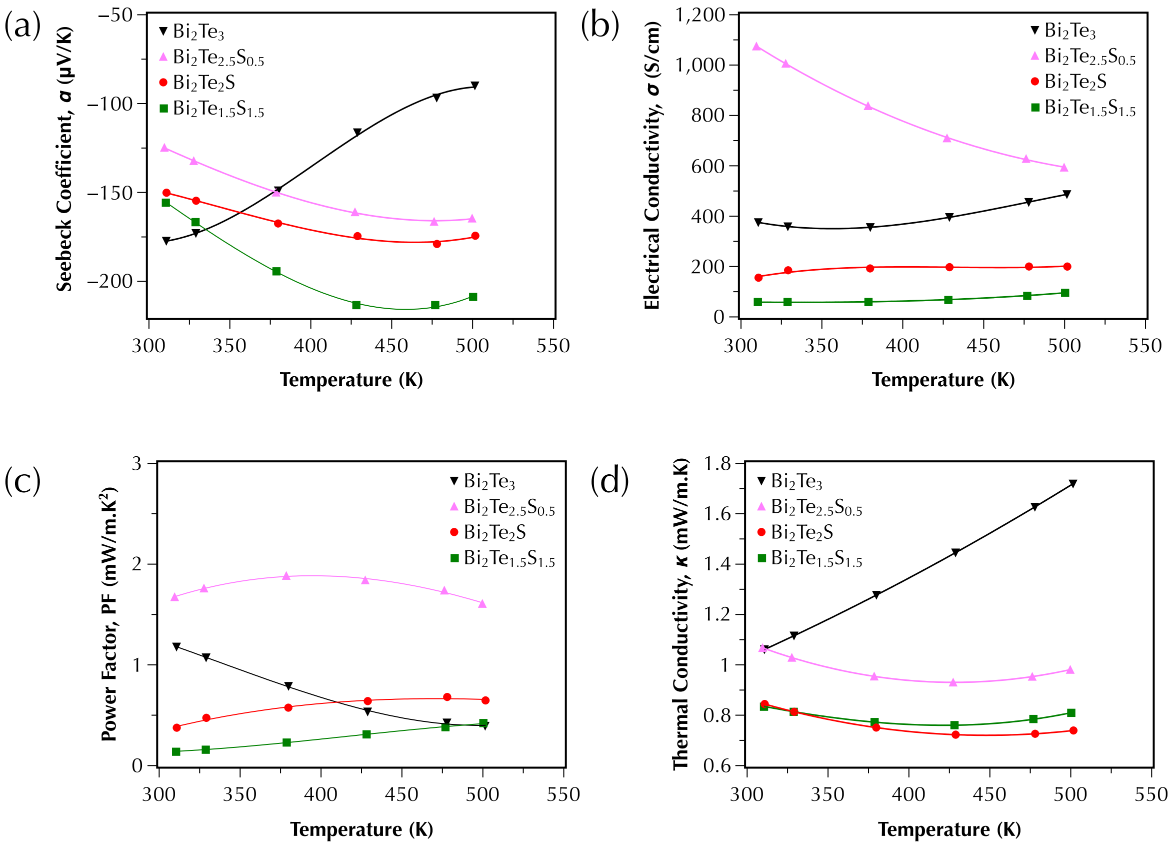

Figure S7. Temperature dependence of (a) the Seebeck coefficient; (b) the electrical conductivity; (c) power factor; and (d) total thermal conductivity of $Bi_2Te_{3-z}S_z$ (z = 0, 0.5, 1.0, 1.5) samples between 300 and 500 K, all properties are measured perpendicular to the sintering direction, except the thermal conductivity which is measured parallel to the sintering direction.

The temperature dependence of the total thermal conductivity, κ, between 300 and 500 K for all $Bi_2Te_{3-z}S_z$ (z = 0, 0.5, 1.0, 1.5) samples is presented in Figure S6(d). The total thermal conductivity of all samples reduces with temperature, except for the single phase Bi_2Te_3 sample. In this case, the total thermal conductivity increases with temperature, in agreement with electrical conductivity and Seebeck coefficient values of this sample, presenting behaviour typical of an intrinsic semiconductor. Although the multiphase samples of $Bi_2Te_{3-z}S_z$ (z = 1 and 1.5) possess low charge carrier concentrations, the bipolar effect is weakly exhibited in these samples. In

general, all multiphase samples demonstrate lower total thermal conductivity than the single phase Bi_2Te_3 sample. The total thermal conductivity of the highly conductive multiphase $Bi_2Te_{2.5}S_{0.5}$ sample is below ~1.1 W/m.K over the temperature range as a result of the finer microstructure (Figure 3).

The multiphase sample of $Bi_2Te_{1.5}S_{1.5}$, comprising the $Bi_{14}Te_{13}S_8$ and Bi_2S_3 phases shows power factors higher than Bi_2S_3 [4,5] and Bi_2Te_2S, even at charge carrier concentrations as low as ~3 $\times 10^{18}$ cm^{-3}. This sample still presents lower power factor and roughly similar total thermal conductivity to multiphase pseudo-binary Bi_2Te_3 – Bi_2S_3 alloys, which contain finer $Bi_{14}Te_{13}S_8$ and Bi_2Te_3 phases.

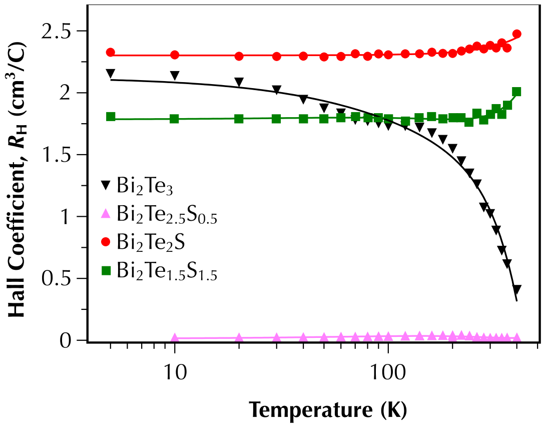

Figure S8. Temperature dependence of the Hall coefficient, R_H, for $Bi_2Te_{3-z}S_z$ (z = 0, 0.5, 1.0, 1.5) samples, between 10 and 400 K, measured on samples cut perpendicular to the pressing direction.

Table S2. Electronic transport parameters perpendicular to the sintering direction at 350 K for $Bi_2Te_{3-z}S_z$ (z = 0, 0.5, 1.0, 1.5) samples.

z	Thermopower (α) (μV/K)	Carrier concentration (n_H) ($\times 10^{19}$ cm^{-3})	Electronic mobility (μ_H) (cm^2/V.s)
0	-173	1.0	234
0.5	-132	30	22
1	-167	0.26	383
1.5	-160	0.32	113

Multiband model

Here, we employed a multiband model[6] for evaluating the thermoelectric properties of the material. The model is described by the following set of equations:

$$\alpha = \frac{\alpha_n \sigma_n + \alpha_p \sigma_p}{\sigma_n + \sigma_p}, \#(1)$$

$$\sigma = \sigma_n + \sigma_p, \#(2)$$

$$\sigma_n = n\mu_n q, \#(3)$$

$$\sigma_p = p\mu_p q. \#(4)$$

where α_n, α_p, σ_n, σ_p are the partial Seebeck coefficient and electrical conductivity of electrons and holes, respectively; μ_n ,μ_p, n, p are, the carrier mobility and carrier density of electrons and holes, respectively; and q is the elementary charge.

The partial Seebeck coefficient, Hall carrier concentration and Hall mobility assuming acoustic phonon scattering can be calculated as follows:

$$\alpha = \frac{k_B}{q}\left(\frac{2F_1(\eta)}{F_0(\eta)} - \eta\right), \#(6)$$

$$n_H = \frac{\left(2m_{\mathrm{DOS}}^* k_B T\right)^{\frac{3}{2}}}{3\pi^2\hbar^3} F_{\frac{1}{2}}(\eta), \#(7)$$

$$\mu_H = \frac{e\pi\hbar^4 \qquad C_l}{\sqrt{2}\,(k_B T)^{\frac{3}{2}}\, E_{\mathrm{def}}^2 m_I^* \left(m_b^*\right)^{\frac{3}{2}}}, \#(8)$$

where η is the reduced Fermi level, m_{DOS}^* is the density of states effective mass, m_b^* is the single valley effective mass, m_I^* is inertial effective mass, E_{def} is the deformation potential for acoustic phonon scattering, and $C_l = \rho v_l^2$is the longitudinal elastic constant of the material that is related to

its density ρ and longitudinal speed of sound v_l in the material. The density of states effective mass is related to the by, $m_{\mathrm{DOS}}^* = N_v^{2/3} m_b^*$ where N_v is the valley degeneracy. In this work, we assumed that the inertial effective mass m_I^* has the same value as the single valley effective mass m_b^* when evaluating Equation (8). The Fermi integral of order j $F_j(\eta)$ is given by the following expression:

$$F_j(\eta) = \int_0^{\infty} \frac{\varepsilon^j}{1 + e^{\varepsilon - \eta}} \mathrm{d}\varepsilon, \#(9)$$

where ε is the reduced energy of the electron state.

These equations are applicable for both electron and holes by noting that $\eta_p = -\eta_n - \epsilon_g$ where η_p is the reduced Fermi level for holes, η_e is the reduced Fermi level for electrons, and $\epsilon_g = E_g/k_bT$ is the reduced bandgap. The measurements of the Seebeck coefficient, carrier concentration, and electrical conductivity were used to estimate the density of states effective mass m_{DOS}^* for both hole and electrons, deformation potential E_{def} of all samples, and reduced Fermi level. The bandgap and longitudinal speed of sound were obtained from literature (Table S3). The valley degeneracy were assumed to be 2 for the conduction band and 6 for the valence band of the two materials [7]. These values were linearly interpolated considering the sample phase composition determined by Rietveld Refinement of the powder diffraction data.

Table S3. Parameters used for modelling

Material	ρ (g/cm^3)	v_l (m/s)	E_g (eV)
Bi_2Te_3	7.8 [8]	2668 [9]	0.15 [10]
$Bi_{14}Te_{13}S_8$	7.55 [11]	2693 [9]	0.28 [12]

Finally, the electronic (κ_e) and bipolar (κ_e) thermal conductivities were estimated as follows:

$$\kappa_e = \sum_{i=n,\ p} L_i \sigma_i T \#(10)$$

$$\kappa_b = \frac{\sigma_p \sigma_n}{\sigma_p + \sigma_n}(\alpha_p - \alpha_n)^2 T \#(11)$$where L is the Lorenz number and is given by:

$$L = \frac{k_B^2 3F_0(\eta)F_2(\eta) - 4F_1^2(\eta)}{q^2 \qquad F_0^2(\eta)} \#(12)$$

References

(1) E37 Committee. *Test Method for Determining Specific Heat Capacity by Differential Scanning Calorimetry*; ASTM International. https://doi.org/10.1520/E1269-11R18.

(2) Witting, I. T.; Chasapis, T. C.; Ricci, F.; Peters, M.; Heinz, N. A.; Hautier, G.; Snyder, G. J. The Thermoelectric Properties of Bismuth Telluride. *Advanced Electronic Materials* **2019**, *5* (6). https://doi.org/10.1002/aelm.201800904.

(3) Fortulan, R.; Aminorroaya Yamini, S.; Nwanebu, C.; Li, S.; Baba, T.; Reece, M. J.; Mori, T. Thermoelectric Performance of N-Type Magnetic Element Doped Bi2S3. *ACS Appl. Energy Mater.* **2022**. https://doi.org/10.1021/acsaem.2c00295.

(4) Tarachand; Okram, G. S.; De, B. K.; Dam, S.; Hussain, S.; Sathe, V.; Deshpande, U.; Lakhani, A.; Kuo, Y.-K. Enhanced Thermoelectric Performance of Novel Reaction Condition-Induced Bi2S3-Bi Nanocomposites. *ACS Appl. Mater. Interfaces* **2020**, *12* (33), 37248–37257. https://doi.org/10.1021/acsami.0c10774.

(5) Wu, Y.; Lou, Q.; Qiu, Y.; Guo, J.; Mei, Z. Y.; Xu, X.; Feng, J.; He, J. Q.; Ge, Z. H. Highly Enhanced Thermoelectric Properties of Nanostructured Bi2S3 Bulk Materials via Carrier Modification and Multi-Scale Phonon Scattering. *Inorg Chem Front* **2019**, *6* (6), 1374–1381. https://doi.org/10.1039/c9qi00213h.

(6) May, A. F.; Snyder, G. J. Introduction to Modeling Thermoelectric Transport at High Temperatures. In *Materials, Preparation, and Characterization in Thermoelectrics*; CRC Press, 2012.

(7) Witting, I. T.; Ricci, F.; Chasapis, T. C.; Hautier, G.; Snyder, G. J. The Thermoelectric Properties of n -Type Bismuth Telluride: Bismuth Selenide Alloys B i 2 T e 3 − x S e x. *Research* **2020**, *2020*, 1–15. https://doi.org/10.34133/2020/4361703.

(8) National Center for Biotechnology Information. *PubChem Compound Summary for CID 6379155*. https://pubchem.ncbi.nlm.nih.gov/compound/6379155 (accessed 2022-03-04).

(9) Tao, Q.; Meng, F.; Zhang, Z.; Cao, Y.; Tang, Y.; Zhao, J.; Su, X.; Uher, C.; Tang, X. The Origin of Ultra-Low Thermal Conductivity of the Bi2Te2S Compound and Boosting the Thermoelectric Performance via Carrier Engineering. *Materials Today Physics* **2021**, *20*, 100472. https://doi.org/10.1016/j.mtphys.2021.100472.

(10) Michiardi, M.; Aguilera, I.; Bianchi, M.; de Carvalho, V. E.; Ladeira, L. O.; Teixeira, N. G.; Soares, E. A.; Friedrich, C.; Blügel, S.; Hofmann, P. Bulk Band Structure of Bi2Te3. *Phys. Rev. B* **2014**, *90* (7), 075105. https://doi.org/10.1103/PhysRevB.90.075105.

(11) Pauling, L. The Formula, Structure, and Chemical Bonding of Tetradymite, Bi14Te13S8, and the Phase Bi14Te15S6. *American Mineralogist* **1975**, *60* (11–12), 994–997.

(12) Wang, L.-L.; Johnson, D. D. Ternary Tetradymite Compounds as Topological Insulators. *Phys. Rev. B* **2011**, *83* (24), 241309. https://doi.org/10.1103/PhysRevB.83.241309.
